# ‘More options…less time’ in the ‘hustle culture’ of ‘generation sensible’: Individualization and drinking decline among twenty‐first century young adults

**DOI:** 10.1111/1468-4446.12964

**Published:** 2022-06-18

**Authors:** Adam Burgess, Henry Yeomans, Laura Fenton

**Affiliations:** ^1^ University of Kent Canterbury UK; ^2^ University of Leeds Leeds UK; ^3^ University of Manchester Manchester UK

**Keywords:** drinking decline, individualization, risk, younger generations

## Abstract

There has been a dramatic decline in alcohol consumption among younger people, including an increase of conscious moderation and abstinence. Change has a generational character, with different cohorts' drinking changing over time from the heavy, embedded pattern among post‐war ‘boomers’ to the more selective habits initiated by ‘millennials’. This is a surprising development in historical terms and has been cast as indicating the emergence of a moderating ‘generation sensible’. It is also coincident with more negative trends, such as young adults worsening mental health. Informed by the perspective of individualization, we consider the decline in youth drinking in the context of generational changes in the lifecourse. We focus upon how recent generations of young people experience greater choice, pressure and a prolonged adolescence, characterized by more limited autonomy. Explored with conscious young moderators through a survey (*N* = 517) and focus groups (*N* = 13), these themes resonated with our sample who appear a self‐conscious generation with significant and open‐ended focus upon maintaining their wellbeing and control. Further, they appear more disembedded from pressure to conform but under greater pressure to perform. The same forces of individualization encouraging moderate drinking may also weigh down upon young people who feel under pressure not only to transform their own lives but feel a burden of responsibility for a damaged, unjust world. The article's originality lies in applying individualization to both generational change and consumption, suggesting this can be usefully done through a focus upon freedom/choice and pressure/performance. It also considers what is regarded as the positive trend of drinking decline alongside, and as related to, negative trends such as greater loneliness and less autonomy among young adults.

## 1 INTRODUCTION

The recent decline of alcohol consumption among younger generations is both remarkable and undisputed (Keyes et al., 2019; Kraus et al., 2020). Different indicators tell a similar story: delayed adolescent initiation, less frequent drinking, less binge drinking and significant numbers drinking little or abstaining altogether. These changes cannot be understood in conventional terms as resulting from changes in pricing or regulation, being consistent across different societies which have experienced different policies and pricing regimes (Kraus et al., 2020). Drinking has fallen the most across Northern Europe and the English‐speaking world but also in the very different socio‐cultural worlds of Japan and Russia, for example. In Russia, as elsewhere, the youngest cohorts are spearheading a more general decline (Radaev & Roschina, 2018, p. 833).

A range of explanatory factors have been mooted. Törrönen et al. (2019) list: changes in parenting style, delaying of adolescence, increased use of social media, changes in gender identities and greater focus upon health and fitness. Kraus et al. (2020) add a reaction against previously high consumption, while the systematic review by Vashishtha et al. (2020) suggests the best evidence supports shifts in parenting practices. Recent accounts look beyond single factors to consider changes within the life course of younger generations (Caluzzi et al., 2022; Pennay et al., 2018). But, despite these valuable contributions, convincing explanations of why generational drinking practices are shifting so markedly—across so many different countries—remain elusive (Room et al., 2019). While different highlighted factors do seem to have—varying degrees of—explanatory value, a framework within which to situate them is lacking. Researchers have called for further research to fill this gap, including qualitative studies that provide deeper explorations of how drinking is perceived and experienced by young people in various social contexts (Pennay et al., 2018).

This article presents original empirical findings from a qualitative study of young people in the UK who regard themselves as moderate or non‐drinkers. It draws upon notions of risk, as preoccupation with possible negative future outcomes (Giddens, 1991), as well as sociological understandings of generations and life course, to provide new insights into how and why Generation Z,^1^ in particular,are choosing to drink little or no alcohol. Specifically, analysis is framed by the notion of individualization. As elucidated by Beck and Beck‐Gernsheim (2002), it attempts to theoretically capture a loosening of ties to social groups and social structures which, in its wake, brings the additional freedom of increased personal choice. At the same time, this enhanced freedom must be negotiated reflexively with an eye upon individual reciprocity and wider social responsibilities—including for public problems or socio‐economic contradictions (Beck & Beck‐Gernsheim, 2002). This dual and reflexive nature of individualization seemed a promising framework for understanding youth drinking decline in a balanced way. Rather than starting from satisfaction with a positive public health outcome, it encouraged a focus upon lived experiences of shifting social structures and social relations to describe and explain changing personal behaviors. Certainly, individualization—through the associated concerns with choice, control and pressure—resonated strongly with our participants. The drink decline, then, is understood less as a logical outcome of positive choices than a behavioral trend reflecting how the phenomenological experiences of contemporary adolescents and young adults have been reshaped by social changes, including extended transitions to adulthood, growing anxiety about the precarity of socio‐economic positions, and the looming threat of environmental catastrophe. As such, this article contributes to our understanding of why drinking is declining through analysis of generational changes to the life course, work, social life and consumption. In doing so, it is distinctive in avoiding a narrow public health or rational choice approach while also demonstrating the application of individualization to an empirical case study and adding to our understanding of the contemporary experience of often fraught contemporary early adulthood.

## 2 GENERATIONAL DRINKING DECLINE, INCREASED ANXIETY AND INDIVIDUALIZATION

The decline in young people's drinking has been closely coincident around the world, with change becoming evident firstly in the USA up to 2000, northern Europe in the early 2000s and western Europe and Australasia by the mid‐2000s (Vashishtha et al., 2020). In Russia, decline was similarly initiated by cohorts born after 1990 (Radaev & Roschina, 2018). American ‘millennials’—as originally defined by Strauss and Howe (2000)—led the way, with the high‐school graduating class of 2000 initiating the shift toward youthful moderation and abstinence. Change was remarkable in both speed and extent, as youth drinking first peaked prior to then plummeting. The UK is an interesting case—not only as an archetypal drinking culture of ‘determined drunkenness’ (Measham & Brain, 2005, p. 268)—but in the transformation of its profile and culture. ‘Binge drinking youth’ were central to a moralization of public discourse about ‘broken Britain’ that continued into the early 2010s (Smith, 2014). Yet, UK figures for having ‘drunk within the last week’ show the biggest fall among young people aged 16–24, from 65% in 1998% to 41% in 2019, while remaining identical for those aged over 65, at 55% (HSE, 2019). In the United States, drinking among older women actually increased, as it fell so dramatically among young people (Keyes et al., 2019). Such patterns reflect wider birth cohort effects over time. The post‐war generation established deeply embedded patterns of higher consumption that persisted to become an assumed norm—though their consumption has fallen too generally, with specific exceptions. The new youth trajectory continues more sharply downwards, meanwhile, with the shift in tastes initiated by millennials appearing to behaviorally harden among the subsequent ‘Generation Z’.

Alcohol consumption remained culturally normalized in Britain and most Western countries across the modern historical period. In these contexts, abstinence and moderation were often understood as ‘signaling behavior’ and ‘the adoption of abstinence became a means to demonstrate good character, industriousness and family values’ (Chrzan, 2013, p. 85), though it is worth noting that abstinence is also associated with a personal history of alcoholism and being in recovery (e.g., Hood, 2003). A body of research has connected subsequent conditions of late modernity to the rise of binge drinking in the UK as, for example, deindustrialized urban areas were regenerated by a night‐time economy which sold the promise of adventure and transgression to receptive young people, culturally steeped in consumer ideals and yearning for some release from daily lives characterized by the monotony of service industry work (e.g., Winlow & Hall, 2006). In this context, studies explored drinking cultures and practices through the identity constructions of young people who drank little or no alcohol, often focusing upon how they justify their decision not to drink to others. It became conventional to present non‐consumption as an exceptional behavior, so far from perceived social norms that skilful negotiation is required (e.g., Conroy & De Visser, 2014; Nairn et al., 2006; Piacentini & Banister, 2009; Supski & Lindsay, 2016). But changes since the 2000s suggest a transformation which has differentiated contemporary drinking practices from the prevalent bingeing habits that preceded them. Increasingly, therefore, researchers have examined non‐drinking young people within the context of an overall decline in youth drinking as, for example, in Månsson et al. (2022) study of young people in Sweden. Drawing together evidence from various international studies, Caluzzi et al. (2022) posit that the drinking decline illustrates a shift away from post‐war norms; non‐drinking is now normalized and drinking is increasingly de‐normalized. This pattern has a cross‐national, even cross‐cultural character. In addition to the patterns from cohort data over time, this coincidence of decline across nations intriguingly points toward *generational change*. At the same time, the trend has intensified over time—beyond only millennials—suggesting a more secular, *supra‐generational trend of late modernity*.

While youth drinking decline has attracted surprisingly little public attention, some commentary in the 2010s identified—and caricatured—it as evidence of an emerging ‘generation sensible’ (BBC, 2018). The Economist (2014) welcomed the ‘staid young’, identifying more responsible and involved parenting as the cause, alongside the more risk‐averse norms and safety culture within which young people have grown up. Whatever the cause, those tracking ‘risky’ behaviors more systematically concur that some trends transcend drinking, especially among ‘Generation Z’. Commenting on the annual Health Survey for England (2016), Gayle (2016) described those born since 2000 to be the ‘most clean‐living generation in recent times’. The report notes that less than 5% of children have smoked, down three‐quarters since 2003, and only 17% admit to ever drinking alcohol, a fall of two‐thirds. They add that even those claiming to eat their ‘five a day’ has doubled in the same period. Research on the lives of younger generations in the U.S. affirms similar trends, understood as a sensibility of moderation (Arnett, 2018).

But there is also a different, more negative trend among the younger generation that attracts greater concern—partly explaining the relative lack of attention for the ‘good news’ of drinking decline. As behaviors like drinking, antisocial behavior and substance use traditionally associated with mental ill‐health are declining, so individualized problems of mental health and self‐harm are actually increasing, demonstrated by both the Millennium Cohort and the Children of the 90s studies, for example, (Patalay & Gage, 2019). Increases are not only an indicator of greater willingness to report problems; both groups were asked identical questions from the age of 14 to minimize differences in how depression was measured. Findings are similar to the pattern of drinking decline, with millennials initiating changes then accelerated by Generation Z; depressive symptoms among the latter are around two thirds higher than among millennials. Trends are similar in the US (Cigna, 2018). Those aged 16–24 years in the UK report also feeling lonely more often than those in older age groups, for example, and younger renters with little trust and sense of belonging to their area are particularly at risk (ONS, 2018). Despite the understandable focus upon the elderly, international studies affirm ‘younger adults are likely to feel more lonely’ (Duffy, 2021, p. 87).

We understand generations here in the basic sense of approximately grouped birth cohorts who have common experiences, behaviors and outlook that to some extent transcend class (Aboim & Vasconcelos, 2014). Approaches such as ‘social generations’ research on youth transitions allows a more holistic view, beyond only class (Chesters et al., 2019; Woodman, 2009). Contemporary sociological understanding tends to emphasize the structural nature of generational problems, in the context of stereotypes about millennials, in particular, which explained away their difficulties in psychological terms; recall the infamous avocado‐on‐toast‐eating millennials of the 2000s, for example! Those using generational frameworks warn of such caricatures, the unlikelihood of precise generational swings and analytical problems of separating cohort, period and life cycle effects (Duffy, 2021; IPSOS Mori, 2020). In short, there is a need to be specific and cautious about generational characteristics and locate these socio‐economically.

Millennials and now Generation Z share a common insecurity rooted in insecure employment and housing opportunities and relatively poor pay (Duffy, 2021). A defining generational characteristic is the end of the expectation that life would get better characteristic of ‘baby‐boomers’ (Petersen, 2021). In an international survey of 10,000 young people, 75% agreed that ‘the future is frightening’, and over half felt they would have fewer opportunities than their parents. Four in ten young people around the world are hesitant to have children as a result (Hickman et al., 2021). This is in stark contrast to the ‘baby‐boomers’ who had the confidence in the conditions of post‐war prosperity to more commonly even have a third child, defining a generation.

Rather than experiencing a short period of insecure employment before transitioning into permanent jobs, young people characteristically go through a ‘new adulthood’ characterized by periods of insecurity, undermining their sense of personal control’ (Chesters et al., 2019). An extended period of adolescence is identified as their defining characteristic, making them ‘not quite’ adult or embodying a new developmental stage of ‘emerging adulthood’ with restricted autonomy (Arnett, 2014; Settersten & Ray, 2010). An important indicator is that 42% of young adults aged between 15 and 34 lived with their parents in the United Kingdom in 2020 compared with 36% in 1996 (Statista, 2021). Expressed proportionally, this constitutes a rise of 17%. Meanwhile, some 18% of American Gen X'ers found themselves still in the family home in 1999, around the age of 27, whereas 31% of millennials were in the same position in 2014. While that now over aged 30 cohort are finally moving out, ‘Gen Z'ers' are even more likely to be stuck at home…’ Duffy (2021: 50), adds that ‘over one million more young adults are living at home in 2019 than 1999, which is an extraordinary change in how we live’. This dimension of individualization casts the centrality of parenting as an explanation for change in a different light (Vashishtha et al., 2020), as not so much being ‘better’ as longer, with the likely inhibiting effect of living with parents stretching into young peoples' twenties. There is an historical comparison here with early modernity up to the 19^th^ Century, and the characteristic experience of a prolonged state of semi‐autonomy for younger people—often in household service—awaiting the opportunity to become self‐sufficient (Stone, 1990).

But we cannot understand drinking decline without also recognizing the positive dimension of generational trends, and here the perspective of individualization is useful (Beck and Beck‐Gernsheim). Pape et al. (2018: 99) suggest drinking decline is ‘embedded in a general trend toward a slower passage into adulthood’. Törrönen et al. (2019: 13) argue that ‘drinking has lost its unquestioned symbolic power as a rite of passage into adulthood. There is less peer pressure to drink and more room for competing activities.’ They further suggest: ‘a hypothesis of the early maturation of young people as more individualized, responsible, reflective, and adult‐like actors than in earlier generations.’ While generational perspectives are more critical (Duffy, 2021), both ‘not quite’ and ‘emerging adult’ perspectives regard these transitional states positively (Arnett, 2014; Settersten & Ray, 2010). Prolonged adolescence allows greater opportunity to mature, greater choice and encourages better decisions as opposed to the compressed experience of baby boomers—even if impacts are uneven and the burden of prolonged support can fall heavily upon poorer families.

The individualization perspective engages this positive aspect more broadly, while simultaneously emphasizing insecurity. Beck and Beck‐Gernsheim (2002) highlight the impact of the cultural revolution of the 1960s, particularly its impact upon women's position, as a far greater range of choices and opportunities compared to the past are at least formally available. Those brought up in its generational aftermath are under increasingly less pressure to conform to established norms, secure a partner and quickly make their own family. They can construct what is termed a ‘choice biography’ where ‘some of the constraints placed on people are breaking down,’ even if ‘the predictability and security that would allow these new options to function as deliberate choices also weaken’ (Woodman, 2009, p. 243). Marriage remains a norm for the Western middle classes, at least, but is typically much later in life after career establishment, and for a variety of individual reasons and fulfillment rather than only family obligation. And the language and emphasis upon individual choice has continued to strengthen with successive generations, even in relation to sexuality. While a majority of Gen Z'ers still see themselves as heterosexual, a third identify as ‘non‐binary’ in the new language of ‘fluidity’ (IPSOS Mori, 2020).

In relation to understanding drinking decline, leisure time need not revolve around the pub or other drink‐centered experiences, as it did for baby boomers. Furthermore, these more individual choices are necessarily more conscious ones. Considered rationally in their own terms—apart from the life course in which they were situated—established practices might make little sense to new generations enjoying different options. Consider the nightclub, for example,—the numbers of which more than halved between 2005 and 2015 (BBC, 2016)—where ‘boomers’ and Gen X'ers once danced, drank and attempted to meet a sexual partner amid deafening music, expensive drinks and unpredictability, but which evidently no longer holds the same appeal. And post‐Internet generations are able to make more informed decisions more broadly, including the harms that might be involved in what previous generations took for granted. Alcohol can now be seen as another drug alongside others which involves risk and benefits—rather than as somehow being more acceptable and harmless, as it tended to be for pre‐millennial generations.

But individualization also engages the more problematic side of changes, drawing out the implications of insecurity for the self. In this reading, late capitalism ‘disembeds’ the individual but is not then able to ‘re‐embed’ in new forms of connection and collectivity; consider the isolation of working in the ‘gig economy’, for example, denied even being an employee, let alone one with rights. New structural and institutional pressures determine greater concern with individual skills and opportunities, pulling away from ties to collective institutions. A competitive, flexible job market requires continually improved performance, and hence retraining, and similar pressures drive young people to perform in a highly individuated fashion in the knowledge economy. New rights and obligations are routinely not addressed to collectivities like community, but instead to the individual. Identity can then be transformed from a given prescribed role into a task, charging each individual with responsibility for performance and the consequences. The process is an open‐ended one without the boundaries or clear demarcation that characterized the prescribed roles and ‘job for life’ of the post‐war boom, leaving a ‘precariat’ defined by economic insecurity and ‘united in decline’ (Beck, 2009, p. 33). Recent accounts of the millennial and post‐millennial condition describe how security remains elusive but they are individually conditioned to respond with only more intense personal endeavor and longer hours. The result can be eventual ‘burn out’ (Petersen, 2021). In the process, the distinction between work and leisure time is blurred. Caluzzi et al. (2021, p. 12) describe how young, moderate drinkers: ‘…adapted their lifestyles, routines and temporal orientations in ways that meant taking ‘time outs’ through alcohol use was secondary to focusing on the future.’ Overall, individualization captures the continual managing of the self in an environment of greater freedom but also of perpetual insecurity and uncertainty.

The economies of Northern Europe and the English‐speaking world which experienced the ‘great risk shift’ toward individualization are also where drinking declines have been striking (Hacker, 2019). Russia experienced an even more sudden shock of exposure to market forces following the collapse of communism, and dramatic drinking decline has accompanied generational and individualized adaptation to the newly competitive environment (Radaev & Roschina, 2018). This is not to suggest a uniform or directly causal process. East Asia has its own pattern of individualization, for example, (Beck, 2010). What is commonly evident is how patterns are shaped by changing socio‐economic circumstances, opportunities and constraints confronting the individual. This does not necessarily mean consistently less consumption. While drinking is low among Chinese emerging adults, it increases once they mature, as ‘they encounter more responsibility and play new roles…especially during social activities where there is a felt pressure to drink’ (Lu et al., 2019). Workplace culture involving obligated heavy drinking remains relatively intact in economically developed East Asian countries (Hansen & Svarverud, 2010). Further, greater women's autonomy in China means they drink slightly more than male emerging adults in one important study with, ‘females developing a stronger independent consciousness, increasing levels of employment, and increasing social activities and communication opportunities’ (Lu et al., 2019). The increases among elderly American women are likely the result of a generation previously culturally constrained enjoying its weakening, as they age and retire in a more liberal environment (Keyes et al., 2019). Overall, however, individualization appears to have driven down consumption as greater pressure, alternative pursuits and health knowledge and concerns have been more influential than the increased choice and access that has come with it. It is to this which we now turn, among our Gen Z research subjects.

## 3 METHODS

To gain insight into generational drinking decline and test whether the duality of individualization resonated with experience we engaged those at the forefront; young people already moderating or abstaining. This was informed particularly by the idea that those subject to individualization are distinctively (self) conscious of doing so as they manage their lives. We explored the subjective practices of moderate or non‐consumption through the use of two methods. Firstly, focus groups were conducted with young people who consciously drink little or no alcohol, recruited from ‘sober’ societies operating within UK universities. Three such societies responded and they were located at universities in southern England and two different parts of Northern England. We conducted four separate focus groups with 13 members of these three societies (10 female; 3 male). The focus groups took place during the coronavirus pandemic and hence were conducted online (via Zoom). Participants were asked about their practices of drinking little or no alcohol as well as their wider recreational activities, understandings of risk and experiences of pressure. They were also invited to discuss perceived generational patterns and differences. We thematically analyzed the resulting data.

Additionally, we conducted an online survey of young people (aged 18–25) who consider themselves moderate or non‐drinkers. The survey was distributed through social media accounts and email distribution lists, and 517 participants completed the questionnaire. The resulting sample is 79% female, 17% male and 1.7% non‐binary. Due to the means of distribution, the sample is skewed toward UK university students and recent graduates. Judging from the last job of their main income‐earning parent, the majority of participants also seemed to be broadly middle class. Results were used to gain insight into perceptions, experiences and general subjective outlook of young moderators in the context of the generational changes identified by data. Basic descriptive statistics were used for analysis. Both focus group and survey data were collected during the early stages of the coronavirus pandemic (April‐July 2020). We instructed participants to answer all questions in relation to their (non) drinking prior to the pandemic, unless otherwise stated. In line with usual ethical practice, pseudonyms are used.

## 4 ALTERNATIVES, CHOICE AND FREEDOM TO EXERCISE THEM

Our research subjects enjoy a strikingly wide range of activities, both alone and with others. As well as the unsurprizing—reading, watching videos and listening to music—nearly 30% of respondents enjoy arts and crafts. Smaller numbers of around 5% list creative hobbies, playing instruments and board games and simply ‘learning new things’. In discussion, Kate is conscious of her alternatives to drinking, listing: baking, shopping, yoga, cinema, bowling, volunteering and seeing friends. Other focus group participants list escape rooms, bowling, roller blading, needle work and (sober) pub quizzes. Tom asserts that their generation is: ‘getting more creative in how to waste their time […] people are thinking of new ways to have fun…’

There is significant practice of direct alternatives to drinking by respondents with 17% using alcohol‐free drinks and mocktails and 31% seeing soft drinks in these terms. In addition, 24% take recreational drugs as a substitute. Alison suggested alternatives can even provide a substitute for one of alcohol's functions as a ‘social lubricant’, avoiding the awkward social moments that alcohol can help overcome. There was recognition of the possibilities opened up; Isabel says that: ‘If you don't drink, you're a bit choosier about what you do…and have more money’ to do so.’ She sees more alternatives to the pub and club even since her sister was her age, 8 years ago, such as trying vegan food.

A strong majority (69%) recognize that they have more choice as to how they spend their time than their parents at the same age. It is the availability of the Internet (52%) that is decisive in their view, followed by greater variety of leisure activities (22%) and more opportunities for travel (13%). As Antony put it:

…because of the internet and being able to do things online we can socially gather without actually gathering, do any kind of activities and that choice allows us to move away…from going to pubs, going to clubs, and drinking.

They also recognize that the place of alcohol in their lives is different, overwhelmingly citing ‘different cultural experiences/expectations’ (68%).

Kate reflects upon the consequences of greater options compared to older generations:

There was less other things for them to do…and I feel like that was very much the coping mechanism of the time. So, if you’ve had a bad day, then you’ll go and drink alcohol, whereas now, if people have days they might be more inclined to go to the gym or do some yoga or some mindfulness or whatever. I feel like people just have more of a broader sense of other ways to cope with things that aren’t necessarily around alcohol.

It is striking that drinking appears here only negatively, as an individualized coping mechanism, rather than also more positively as reducing social inhibition and interacting with others.

The young respondents recognized their parents' generation as drinking in specific contexts, some of them distinct from their own: de‐stressing after work (16.8%), to accompany a meal (19.3%) and watching television (10%). More similar to their own habits came socializing with friends (23%) and at special occasions (24.4%). Asked about times and experiences they associate with parental generation drinking, they single out ‘weekend treat/time off of work’ (31.8%). It is reasonable to suppose that it is because post‐work time conditioned drinking is different from their own that they recognize it so readily in others. A majority thought that drinking—including to excess—was ‘associated with becoming adult’ when their parents were their age, but were equally divided over whether it was now associated with being a teenager, adult or neither. Drinking may have an unclear status amid a generally unclear extended adolescence with few clear markers of transition (Törrönen et al., 2019).

The contrast with the more compressed but apparently more certain life course of previous generations and connection to drinking culture was clear to some. Hannah describes the experiences of her father who settled to work and marriage early on and drank heavily around a routine job with little care for his welfare. There was consensus that they have more opportunities than their parents, particularly from not having a family at a young age. As to whether they think their habits will change as they reach their parents' age, they are evenly split with an unusually high remainder (24%) not knowing. Such uncertainty may reflect the difficulty of envisaging themselves replicating their parents' life course, even if later.

Some interviewees stressed generational difference was not simply the availability of alternatives, but the freedom to enjoy them with less judgment or stigma, as in the case of Chinese and American women, above (Keyes et al., 2019; Lu et al., 2019)—including drinking too much or none at all. They could be thought of as ‘generation diversity’, according to Kate. As Tom put it:

Our generation tends to, on all fronts be a bit more of accepting of who you are and…want to be, so…there’s a lot less pressure for people to drink…Like, sod it then, why bother if no one’s even pushing me?

At the same time, separated out as an individual rather than social experience and posed as a choice, getting used to regular drinking can appear strange to our moderators. As Ivy put it: ‘forcing themselves to drink something until they like it, it's like why would you do that?‘. As a conscious individual choice, it has become de‐normalized (Caluzzi et al., 2022).

## 5 PRESSURE, ANXIETY…CONTROL

Greater choice and freedom may be liberating but also create greater pressure (Beck & Beck‐Gernsheim, 2002). More things to do suggests you should be doing them. As Alison put it:

… They drank what would probably be considered a normal amount then. But…there was no other options and they weren’t bothered about doing anything else, whereas now there is always something I can be doing.

The issue of pressure comes across as the most readily recognized and universal across the focus groups. Education figures prominently and the need to do well in every aspect of life—while it being unclear how this might be achieved in such an open and competitive environment. Almost twice as many survey respondents felt ‘under pressure to perform as a teenager/young adult’ as did not (62%/32%). Responses to the ways in which this pressure was experienced were evenly high. Academic achievement was highest (22%) followed by ‘deciding upon a career’ (20%), growing up (17%), being responsible for own mental wellbeing (16%), staying physically healthy (15%) and finding a life partner (10%).

A striking 70% affirmed their generation to be under greater pressure than their parents'. For Courtney:

…with my parents, it was very much just finish school, get a job, whereas my mum’s always been like, “Do what you want but make sure you use your education to the best of your ability,” like use the opportunities that they didn’t have.

The supposedly empowering contemporary parental injunction that ‘you can do anything, darling’ can be experienced as endless pressure, as much as endless opportunity, driving eventual ‘burnout’ (Petersen, 2021).

From research with young people in Australia, Caluzzi et al. (2021) suggest that a perceived need to use free time productively is connected to drinking decline. Some of our respondents' accounts lent weight to this view. Alison referred to a ‘hustle culture’ among Generation Z, meaning:

I think we have more options, but I also think we have less time. We have this mentality of sitting around and talking to people isn’t worth it. I mean, it is…but…we have it so ingrained in us that everything we do has to have like a productive outcome…you have to be constantly looking forward.

The most tangible pressure is the burden of university debt, mentioned as directly curbing inclinations toward hedonism. Contemporary university life more broadly reflects the range of individualized pressures; as Kate put it:

…you need to be doing everything. I remember my first lecture at uni…we’d only been there… two days, they were like right…so you need to book an appointment with the careers centre and you need to get yourself some voluntary experience and then there's loads of part time jobs…. Also there's these societies you need to join. You should write for the psychology newspaper, and there's this peer mentoring scheme that you need to join and it was literally just like, oh my God, I just want to do my degree’

This was counter‐posed generationally to the experience of their academics from a previous, less pressured generation:

…the amount of lecturers who are in their sixties who…got 2:1s and did no experience and got on PhDs and got lecturing jobs afterwards, whereas nowadays… (Isabel)

But pressures are far from confined to education. Kate continued:

Oh my God, yeah…(we are) Generation Do‐It‐All because you’re expected to be at uni, have a part time job, do volunteering, have a boyfriend, have this buzzing social life, work out every day, have this like nice‐toned body, have the perfect diet, have a nice car, like just so much and I feel like social media doesn’t help because everyone just seems to have their lives together.

Hannah explained a direct link between the extended, work‐related pressure to perform and choice of sober social activity. Social drinking is associated with performance pressure as opposed to the ideal of a gentler night in with friends, centered on scrabble or monopoly (notably still competitive activities!). Sophie said:

…that increase of pressure [between our and our parents' generation] means that we maybe wouldn’t drink as much because we don’t have the time or it feels like time wasted…

This is not to say that pressure is responded to by all with moderation. Others cope in more traditional ways by partying and getting drunk, even if: ‘maybe they'll regret it in the morning when they're hungover at a 9a.m. seminar’, according to Sophie.

Even lockdown provided no relief, according to Isabel:

I feel like employers expect you to have done loads, so if you haven’t, they’re like “What did you do with your time?” Everyone’s coming out of lockdown, feeling like they should have developed a new skill and become a master in something…

Pressure determines an emphasis upon the need to maintain *control*, a recurrent theme from our respondents and participants and related to time management (Caluzzi et al., 2021). Alongside the problems drinking poses for maintaining good health, it is the challenge to (self) control that damns it in the eyes of young moderators. A high 60% of respondents associate drinking with the loss of control, almost twice those that did not. Focus group participants agreed. For example, Sophie said: ‘Definitely…one of my key reasons…I don't know if I can trust myself with alcohol, and it's not something that I'd want to risk losing control….’

A 71% majority affirmed there are consequences to drinking that concern them; it is not simply a pleasure or release to be enjoyed in the moment, as it might more easily have been in the past. Future considerations—risks—overshadow the present (Giddens, 1991). Which consequences were to the fore? The most significant was ‘health issues’ (21%) followed by ‘loss of control’ (13%) and change in behavior/embarrassment (10%). More specific consequences of hangover, loss of memory/blackouts, concerns about addiction and increased vulnerability were also highlighted by over 5% each. These consequences heavily influence the decision to drink or not; among those who specified concerns, 34% see them as ‘extremely important’, 40% ‘very important’ and 21% as ‘slightly important’. The significance attached to the consequences of intoxication contrasts sharply with the idea of a ‘culture of intoxication’ in which young people widely use alcohol to lower inhibitions (Measham & Brain, 2005), and echoes Caluzzi et al.’s (2020b) claim that many young people in countries where youth drinking has declined no longer understand the loss of inhibition as pleasurable.

## 6 HEALTH, RISK AND ‘GENERATION SENSIBLE’?

Loss of control may be undesirable in itself but it also suggests risk to the individual. Asked specifically about vulnerability to crime—the sharpest end of concern—a strong majority of 66% agreed that they were concerned about alcohol's role. In this context, the predominantly female sample indicated that it was either ‘somewhat’, ‘quite’ or ‘very’ important in their decision to moderate drinking, with only 20% disagreeing. Being ‘taken advantage of’ is ‘in the head’ and ‘always a concern’ of Ivy and Elise. The connection has been made so strongly in the university context that the two are synonymous; James (male): ‘associate(s) alcohol with rape culture as it were…’.

Given a wide range of potentially concerning consequences of drinking, general ‘health issues’ were predominant, however, with over 70% considering them ‘extremely’, ‘very’ or ‘quite’ important. Respondents see the health risks of alcohol as high. Over 90% associated ‘particular health risks with drinking alcohol’. In fact, they are seen as more significant than the consensus in the medical community. A majority of 64% see the risks of alcohol as equivalent to those of smoking, regardless of the unique and powerful connection of smoking to lung cancer which has no alcohol equivalent. Only 8% declared themselves ‘unsure’. This strong perception of health risk partially corroborates Caluzzi et al.'s (2020a) argument that the decline of youth drinking is shaped by the internalization of ‘healthism’—a socio‐cultural practice in which health becomes “a primary… focus for the definition and achievement of wellbeing” (Crawford, 1980, p. 368). Indeed, the moral imperative of caring for one's health and wellbeing is an important component of contemporary individualism (Brandt & Rozin, 1997).

But at the same time, our respondents offered informed criticism of alcohol messaging and awareness that moderate consumption is unlikely to be causing harm. There is balanced risk awareness rather than simple aversion:

But the more sort of conventional health issues, like…the liver and kidney damage and weight gain and things like that…don’t particularly bother me…the amount of diet coke that I drink probably makes up for that. (Alison, small northern university)

Hannah was smartly critical of health messaging failing to promote sensible harm reduction:

There’s no like, “Experiment a little bit, do everything in moderation, just to like try it, to like quench your curiosity. Just don’t do it in excess.”

There was significant uncertainty evident when respondents were asked to compare other risks, which may reflect how ‘health’ concerns are quite general—an ‘ism’ (Crawford, 1980)—and bleed into lifestyle and ethics (Brandt & Rozin, 1997). Large majorities were ‘unsure’ whether risks posed by air pollution, driving, eating meat and sugar were more significant than drinking. Many of the same respondents indicated cutting down on these things, however, suggesting other drivers at play—even a generic working principle of moderation. A broader sense of wellbeing and mental health emerged as a more important in focus groups. When it came to the telling issue of actual behavior to manage risks, a majority of 40% said they took action to avoid or control certain risky activities, with others split between not knowing and rejecting—suggesting particular risks are significant but not over‐riding factors. Smoking and recreational drugs were each avoided by 30%. The range of avoided behavior and sometimes unclear connection to harm suggests an issue of control more than risk per se.

There is little sense from our data that young moderators are compulsively risk averse; that they are indeed like the mythical millennial ‘snowflakes’ (Duffy, 2021). While anxious and health conscious, risk figures more strongly in the sense suggested by Giddens (1991), as a focused orientation toward the future. Individualized pressures determine little time for short‐term pleasures (Caluzzi et al., 2021). Kate expressed bemusement at other students' spending on nights out drinking, asking:

…why? They don’t even remember anything about that night because they were obviously too drunk to remember, so I just think you could have literally just thrown that money down the drain, like what is the point?’

Turning to the ‘generation sensible’ notion (BBC, 2018), there was considerable self‐identification among our moderators with a slight majority (42.2%) recognizing themselves in the term against 39.1% who didn't. A majority (59%) didn't think they were representative of their generation, with 53.4% instead seeing themselves as part of a significant sub‐set, with 14.8% unsure.

**FIGURE 1:**
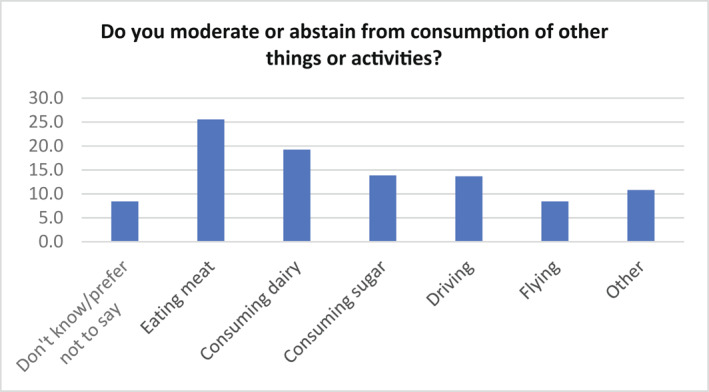
Other things which survey participants avoid

There was striking affirmation for the idea of a moderating, consciously ‘sensible’ generation—or sub‐set—in answers to moderating/abstaining from other things (Figure 1). There is a range of quite evenly spread responses across things and activities. Respondents appear to have had little trouble in seeing (not) flying and driving in the same terms as (not) eating sugar or dairy. An orientation against (excessive) consumption appears as a characteristic comparable to the ‘qualified materialism’ of boomers (Leach et al., 2013).

Yet the ‘sensible’ label is an odd characterization unless reframed. Parental influence was not judged to be very significant by our respondents and yet it is their parents' greater time and input that is central to the idea of ‘generation sensible’ and, indeed, drinking decline as a whole (Economist, 2014; Vashishtha et al., 2020). A strong majority do not think their drinking habits would have changed if their parents had drunk less and a clear majority only see them as having shaped drinking habits a little, or, more frequently, not at all. Further, this is an ‘anything goes’ generation, something that doesn't readily square with caution. Previously deviant and sub‐cultural behaviors like drug taking have been normalized, which began in the 1990s (Parker et al., 1998). As Kate put it: ‘I think most of my friends probably have like a drug dealer's contact in their phone for like weed or ecstasy or anything like that…’

Instead, some explain that they see the ‘sensible’ dimension as not related to being intuitively risk averse, but feeling responsible for a worrying, uncertain future. Tom explains:

…I don’t think it’s about risk, I think it’s about we’re more active on wanting to see change, we don’t drive cars as much because we’re more concerned about the environment, same with food, we don’t drink because we know it leads to alcoholism, and we’re more active within just various things, the BLM movement that’s happening right all around us, we don’t really want to be quiet about things anymore.

Jennifer explains that they are:

hyper‐aware of the world as it is and the problems with climate change and the political climate in the world creating pressure on us to be doing our best to be making the world a better place, rather than just like living for the moment and having fun…almost like a scared generation, or a generation who feel the pressure to be not looking out only for ourselves but to be like doing the best we can do for everybody else.

Sophie adds that that the same pressure is felt by people of her generation who are not moderate or non‐drinkers (Caluzzi et al., 2021).

## 7 CONCLUSIONS: MORE OPTIONS, BUT MORE PRESSURE

Our themes resonated with both survey respondents and interviewees and in this sense framing research around the individualization perspective was useful, stimulating interesting reflection and helping make sense of how their habits differ so dramatically from post‐war generations. Young people have a far more meaningful choice to no longer drink and instead do other things previously unavailable. But greater choice is not celebrated, and perhaps not only because they have become accustomed to it. Following the explanation of Beck's ‘choice biography’ by Woodman (2009), there is indeed greater choice, opportunity and even compulsion to ‘make a life of one's own’, but those choices are made—echoing Marx—‘not under circumstances of their choosing’. Their lives are as overshadowed by economic insecurity as post‐war generations were defined by greater security, and the individualized pressure to perform under such circumstances seems as significant in turning young people away from drinking as greater choice. And life does appear to present itself as a project of the self in conditions of uncertainty, such as through the focus upon maintaining good health and the imperative to always stay in control that both further direct them away from drinking.

Generational distinctions are characteristically marked by defining external events rather than only a purported proclivity such as risk aversion (Duffy, 2021). Thus the ‘silent generation’ born in the interwar period who endured the privations of depression and war and the subsequent ‘baby‐boomers’ who enjoyed the subsequent economic take‐off. ‘Generation Z’ appear marked by external pressures of expectation to make a damaged world a better place while simultaneously realizing their potential to ‘be whoever they want to be’. While not having to conform, they do feel pressured to perform—without end, related to the notion of always bringing one's ‘best self’ to the table (Fry, 2011). As interviews with Gen Z across Europe were sub‐titled, they are a generation with ‘so many revolutions to lead’ (Guardian, 2021). Future burdens such as climate change appear to determine a more general orientation against ‘excessive’ consumption, be that of meat, dairy or other things (Hickman et al., 2021). There is some sense then in which—at least among this sample—they are a ‘generation sensible’ with a wider attitudinal disposition toward moderation. ‘Boomers’, by contrast, particularly later‐born ones, are a ‘bridging generation’ between the conspicuous consumption of their (Generation X) children and the ‘make do and mend’ of their parents (Leach et al., 2013).

The contemporary ‘young adults’ in our sample show themselves to be also highly self‐conscious; even able to recognize the distinctiveness of their circumstances and greater choices compared to their parents' generation. Adopting a generational lens enables us to see how for this pioneering group of conscious moderators, drinking has become as disembedded from their lives as it was deeply embedded in those of the post‐war generations. Younger peoples' drinking decline thus does not seem so ‘remarkable’ having developed a sense of their lifeworld through this research. Rather, it seems an unsurprizing response to a very different life‐course and (global) environment in which they find themselves. Both the distinctive pressures and opportunities of millennial and post‐millennial generations direct them away from the more fixed and narrow routines of their parents. Their lives are characterized by choices, pressures and information rather than compressed adolescence, familial responsibilities, restrictive values, and work routines and escape from them. In fact, what *now seems odd is how the heavy drinking of the post‐war generations that accompanied the post‐war life‐course still acts as the norm from which the habits of current younger generations are judged to deviate*.

The decline in drinking—especially youth drinking—is uncritically welcomed in most research and, judged by likely physical health outcomes alone, might constitute a positive trend. However, examined from a broader sociological perspective, our findings suggest it is not a simple good news story. It may be connected to anxieties and uncertainties rooted in intensifying social conditions of precarity and pressure to perform in education and other institutional settings. While others have linked changing drinking habits to changes in parental practice (Vashishtha et al., 2020) or the rise of healthism (Caluzzi et al., 2020a), we found it to also be linked to a wider set of socio‐economic and cultural conditions that have shaped young people's lifeworld. Our contribution is to propose that individualization is a useful lens to make sense of these generational changes and has explanatory value in relation to drinking decline. Enhanced leisure opportunities—in comparison to previous generations—and intense individual pressure and responsibility have profound impacts on the lived experiences of Millennials and then Generation Z and shape their consumption practices. Ultimately, the process of individualization may transform various public problems—from climate change to anxiety about educational performance—into personal routines of moderate or non‐drinking.

Finally, this study is explorative rather than definitive. While our conclusions are broadly drawn they are also tentative, intended to stimulate further research and reflection. We also acknowledge that the study—like the individualization thesis itself—is skewed toward what has changed over what remains continuous, and in this sense is one sided and open to charges of overstatement. Further, the sample was also skewed heavily toward middle class females—though they self‐selected and this does appear to be the profile of those at the forefront of the generational changes we describe. Unsurprisingly, a majority were also university‐educated and this is relevant to understanding the particular ways in which pressures and performance are experienced and recalled—particularly sharply in the additional burdens of debt and cv‐building, alongside study. It is a question for further research to examine how much we can generalize to others such as young working class males ‐though the overall data suggest that most groups are drinking less and this is likely to continue. Alcohol remains a drug bound up with human sociability and perhaps it is no surprise that in an age of increasing individualism and individualization it is also increasingly less embedded in our lives.

## CONFLICT OF INTEREST

There is no conflict of interest.

## ETHICS STATEMENT

Ethical approval was given by SRC Ethics Panel of the University of Kent School of Social Policy, Sociology and Social Research (reference: SRCEA id 258).

## Data Availability

Raw data were generated at the University of Kent. Derived data supporting the findings of this study are available from the corresponding author on request. The data that support the findings of this study are available on request from the corresponding author.

## ENDNOTE

^1^
The consensus is that ‘baby‐boomers’ are those born between 1945 and 65; ‘Generation X’ between 1966 and 79; ‘millennials’ between 1980 and 95. There is less agreement over the emerging boundary between ‘millennials’ and ‘Generation Z’ where it is either those born between 1996 and 2010; born after 1997 or born after 2000 (Duffy, 2021, p. 19).

## References

[bjos12964-bib-0001] Aboim, S. , & Vasconcelos, P. (2014). From political to social generations: A critical reappraisal of Mannheim’s classical approach. European Journal of Social Theory, 17(2), 165–183.

[bjos12964-bib-0002] Arnett, J. (2014). Emerging adulthood. Oxford University Press.

[bjos12964-bib-0003] Arnett, J. (2018). Getting better all the time: Trends in risk behavior among American adolescents since 1990. Archives of Scientific Psychology, 6, 87–95. 10.1037/arc0000046

[bjos12964-bib-0004] BBC . (2016). Whatever happened to the great British nightclub? 16 March. Available at: https://www.bbc.co.uk/news/magazine‐35820320

[bjos12964-bib-0005] BBC . (2018). Generation sensible in five charts. (19 July). Available at: https://www.bbc.co.uk/news/44880278

[bjos12964-bib-0006] Beck, U. (2010). Foreword: Varieties of individualization. In M. Hansen & R. Svarverud (Eds.), IChina: The rise of the individual in modern Chinese society. NIAS Press.

[bjos12964-bib-0007] Beck, U. , & Beck‐Gernsheim, E. (2002). Individualization: Institutionalized individualism and its social and political consequences. Sage.

[bjos12964-bib-0008] Beck, U. , & Beck‐Gernsheim, E. (2009). Global generations and the trap of methodological nationalism. European Sociological Review, 25(1), 25–36.

[bjos12964-bib-0009] Brandt, A. , & Rozin, P. (1997). Morality and health. Routledge.

[bjos12964-bib-0010] Caluzzi, G. , Livingston, M. , Holmes, J. , MacLean, S. , Lubman, D. , Dietze, P. , Vashishtha, R. , Herring, R. , & Pennay, A. (2022). Declining drinking among adolescents: Are we seeing a de‐normalisation of drinking and a normalisation of non‐drinking? Addiction, 117(5), 1204–1212. 34159676 10.1111/add.15611 PMC7614939

[bjos12964-bib-0011] Caluzzi, G. , MacLean, S. , & Pennay, A. (2020a). ‘No one associated alcohol with being in good health’: Health and wellbeing as imperatives to manage alcohol use for young people. Sociology of Health & Illness, 43(2), 493–509. 10.1111/1467-9566.13237 33635553

[bjos12964-bib-0012] Caluzzi, G. , MacLean, S. , & Pennay, A. (2020b). Re‐configured pleasures: How young people feel good through abstaining or moderating their drinking. International Journal of Drug Policy, 77, 102709. 10.1016/j.drugpo.2020.102709 32120247

[bjos12964-bib-0013] Caluzzi, G. , Pennay, A. , MacLean, S. , & Woodman, D. (2021). No time for a ‘time out’? Managing time around (non) drinking. Sociology early view, 56(1), 21–37. 10.1177/00380385211008370

[bjos12964-bib-0014] Chesters, J. , Smith, J. , Cuervo, H. , Laughland‐Booy, J. , Wyn, J. , Skrbis, Z. , & Woodman, D. (2019). Young adulthood in uncertain times: The association between sense of personal control and employment, education, personal relationships and health. Journal of Sociology, 55(2), 389–408.

[bjos12964-bib-0015] Chrzan, J. (2013). Alcohol: Social drinking in cultural context. Routledge.

[bjos12964-bib-0016] Cigna . (2018). Cigna loneliness index. Available at: https://www.multivu.com/players/English/8294451‐cigna‐us‐loneliness‐survey/

[bjos12964-bib-0017] Conroy, D. , & De Visser, R. (2014). Being a non‐drinking student: An interpretative phenomenological analysis. Psychology and Health, 29(5), 536–551. 24245802 10.1080/08870446.2013.866673

[bjos12964-bib-0018] Crawford, R. (1980). Healthism and the medicalization of everyday life. International Journal of Health Services, 10(3), 365–388. 7419309 10.2190/3H2H-3XJN-3KAY-G9NY

[bjos12964-bib-0019] Duffy, B. (2021). Generations. Atlantic Books.

[bjos12964-bib-0020] Economist . (2014). The Staid Young. Oh, you pretty things. (12 July). Available at: https://www.economist.com/briefing/2014/07/12/oh‐you‐pretty‐things

[bjos12964-bib-0021] Fry, M. L. (2011). ‘Discourses of consumers’ alcohol resistant identities. Journal of Nonprofit & Public Sector Marketing, 23(4), 348–366.

[bjos12964-bib-0022] Gayle, D. (2016). Smoking and drinking among young people at lowest level on record. (15 December). The Guardian. Available at: https://www.theguardian.com/society/2016/dec/15/smoking‐drinking‐young‐people‐lowest‐level‐on‐record‐england

[bjos12964-bib-0023] Giddens, A. (1991). The consequences of modernity. Stanford University Press.

[bjos12964-bib-0024] Guardian (2021). Europe’s gen Z in their own words. (2 June). Available at: https://www.theguardian.com/world/ng‐interactive/2021/jun/02/so‐many‐revolutions‐to‐lead‐europe‐generation‐z‐on‐their‐post‐covid‐future

[bjos12964-bib-0025] Hacker, J. (2019). The great risk shift. Oxford University Press.

[bjos12964-bib-0026] Hansen, M. , & Svarverud, R. (2010). IChina: The rise of the individual in modern Chinese society. NIAS Press.

[bjos12964-bib-0027] Health Survey for England . (2016). NHS digital. Available at: https://digital.nhs.uk/data‐and‐information/publications/statistical/health‐survey‐for‐england/health‐survey‐for‐england‐2015

[bjos12964-bib-0028] Hickman, C. , Marks, E., Pihkala, P., Clayton, S., Lewandowski, E., Mayall, E., Wray, B., Mellor, C., & van Susteren, L. (2021). Young people's voices on climate anxiety, government betrayal and moral injury: A global phenomenon. The Lancet. (preprint) Available at: https://papers.ssrn.com/sol3/papers.cfm?abstract_id=3918955 10.1016/S2542-5196(21)00278-3 34895496

[bjos12964-bib-0029] Hood, C. D. (2003). Women in recovery from alcoholism: The place of leisure. Leisure Sciences, 25(1), 51–79.

[bjos12964-bib-0030] HSE . (2019). Health survey for England: Alcohol. Available at: http://healthsurvey.hscic.gov.uk/data‐visualisation/data‐visualisation/explore‐the‐trends/alcohol.aspx

[bjos12964-bib-0031] IPSOS Mori . (2020). Beyond binary: The lives and choices of generation Z. IPSOS Mori.

[bjos12964-bib-0032] Keyes, K. M. , Jager, J. , Mal‐Sarkar, T. , Patrick, M. E. , Rutherford, C. , & Hasin, D. (2019). Is there a recent epidemic of women's drinking? A critical review of national studies. Alcoholism: Clinical and Experimental Research, 43(7), 1344–1359. 31074877 10.1111/acer.14082 PMC6602861

[bjos12964-bib-0033] Kraus, L. , Room, R. , Livingston, M. , Pennay, A. , Holmes, J. , & Torronen, J. (2020). Long waves of consumption or a unique social generation? Exploring recent declines in youth drinking. Addiction Research and Theory, 28(3), 183–193. 10.1080/16066359.2019.1629426 PMC7594162 33132794

[bjos12964-bib-0034] Leach, R. , Phillipson, C. , Biggs, S. , & Money, A. (2013). Baby boomers, consumption and social change: The bridging generation? International Review of Sociology, 23(1), 104–122.

[bjos12964-bib-0035] Lu, W. , Xu, J. , Taylor, A. W. , Bewick, B. M. , Fu, Z. , Wu, N. , Qian, L. , & Yin, P. (2019). Analysis of the alcohol drinking behavior and influencing factors among emerging adults and young adults: A cross‐sectional study in wuhan, China. BMC Public Health, 19(458). 10.1186/s12889-019-6831-0 PMC6492408 31039783

[bjos12964-bib-0036] Månsson, J. , Samuelsson, E. , & Törrönen, J. (2022). Doing adulthood ‐ Doing alcohol: What happens when the “sober generation” grows up? Journal of Youth Studies, 25(1), 84–99.

[bjos12964-bib-0037] Measham, F. , & Brain, K. (2005). ‘Binge’ drinking, British alcohol policy and the new culture of intoxication. Crime, Media, Culture, 1, 262–283.

[bjos12964-bib-0038] Nairn, K. , Higgins, J. , Thompson, B. , Anderson, M. , & Fu, N. (2006). “It’s just like the teenage stereotype, you go out and drink and stuff”: Hearing from young people who don’t drink. Youth Studies, 9(3), 287–304.

[bjos12964-bib-0039] ONS . (2018). Loneliness ‐ What characteristics and circumstances are associated with feeling lonely? Office for National Statistics.

[bjos12964-bib-0040] Pape, H. , Rossow, I. , & Brunborg, G. (2018). Adolescents drink less: How, who and why? A review of the recent research literature. Drug and Alcohol Review, 37, 98–114. 10.1111/dar.12695 29573020

[bjos12964-bib-0041] Parker, H. J. , Aldridge, J. , & Measham, F. (1998). Illegal leisure: The normalization of adolescent recreational drug use. Routledge.

[bjos12964-bib-0042] Patalay, P. , & Gage, S. (2019). Changes in millennial adolescent mental health and health‐related behaviours over 10 years: A population cohort comparison study. International Journal of Epidemiology, 48(5), 1650–1664. 30815691 10.1093/ije/dyz006 PMC6904321

[bjos12964-bib-0043] Pennay, A. , Holmes, J. , Torronen, J. , Livingston, M. , Kraus, L. , & Room, R. (2018). Researching the decline in adolescent drinking: The need for a global and generational approach. Drug and Alcohol Review, 37(S1), S115–S119. 29431253 10.1111/dar.12664

[bjos12964-bib-0044] Petersen, A. H. (2021). Can’t even: How millennials became the burn out generation. Vintage.

[bjos12964-bib-0045] Piacentini, M. , & Banister, E. (2009). Managing anti‐consumption in an excessive drinking culture. Journal of Business Research, 62, 279–288. 10.1016/j.jbusres.2008.01.035

[bjos12964-bib-0046] Radaev, V. , & Roschina, Y. (2018). Young cohorts of Russians drink less: Age–period–cohort modelling of alcohol use prevalence 1994–2016. Addiction, 114, 823–835. 10.1111/add.14535 30552861

[bjos12964-bib-0047] Room, R. , Greenfield, T. K. , Holmes, J. , Kraus, L. , Livingston, M. , Pennay, A. , & Torronen, J. (2019). Supranational changes in drinking patterns: Factors in explanatory models of substantial and parallel social change. Addiction Research and Theory, 28(6), 467–473. 33132794 10.1080/16066359.2019.1689963 PMC7594162

[bjos12964-bib-0048] Settersten, R. , & Ray, B. (2010). Not quite adult. Bantam.

[bjos12964-bib-0049] Smith, O. (2014). Binge Britain. In Contemporary adulthood and the night‐time economy. Leisure studies in a global era. Palgrave Macmillan.

[bjos12964-bib-0050] Statista (2021). Percentage of young adults living with their parents UK 1996‐2020. Available at: https://www.statista.com/statistics/285339/percentage‐of‐young‐adults‐living‐with‐parents‐uk/

[bjos12964-bib-0051] Stone, L. (1990). The family, sex & marriage in England 1500‐1800. Penguin.

[bjos12964-bib-0052] Strauss, W. , & Howe, N. (2000). Millennials rising: The next great generation. Vintage Books.

[bjos12964-bib-0053] Supski, S. , & Lindsay, J. (2016). “There’s something wrong with you”: How young people choose abstinence in a heavy drinking culture. Young, 25(4), 1–16.

[bjos12964-bib-0054] Törrönen, J. , Roumeliotis, F. , Samuelsson, E. , Kraus, L. , & Room, R. (2019). Why are young people drink less than earlier? Identifying and specifying social mechanisms with a pragmatist approach. International Journal of Drug Policy, 64, 13–20. 10.1016/j.drugpo.2018.12.001 30544091

[bjos12964-bib-0055] Vashishtha, R. , Livingston, M. , Pennay, A. , Dietze, P. , MacLean, S. , Holmes, J. , Herring, R. , Caluzzi, G. , & Lubman, D. I. (2020). Why is adolescent drinking declining? A systematic review and narrative synthesis. Addiction Research and Theory, 28(4), 275–288. 10.1080/16066359.2019.1663831

[bjos12964-bib-0056] Winlow, S. , & Hall, S. (2006). Violent night: Urban leisure and contemporary culture. Berg.

[bjos12964-bib-0057] Woodman, D. (2009). The mysterious case of the pervasive choice biography: Ulrich Beck, structure/agency, and the middling state of theory in the sociology of youth. Journal of Youth Studies, 12(3), 243–256.

